# Supplementary material for: Chickpea-Derived Prebiotic Substances Trigger Biofilm Formation by Bacillus subtilis
Source: Nutrients. 2021 Nov 25;13(12):4228. doi: 10.3390/nu13124228 (PMC8704855; doi:10.3390/nu13124228)
Supplement: Supplementary file 1 [file nutrients-13-04228-s001.zip › nutrients-1446569-supplementary.pdf]

Supplementary Data

Chickpea Derived Prebiotic Substances Trigger Biofilm Formation by *Bacillus subtilis*

Yaa Serwaah Amoah[1,2], Satish Kumar Rajasekharan[1], Ram Reifen[2] and Moshe Shemesh[1,*]

1 Department of Food Sciences, Institute for Postharvest Technology and Food Sciences, Agricultural Research Organization (ARO), Volcani Institute, Rishon LeZion 7528809, Israel.
2 The Robert H. Smith Faculty of Agriculture, Food and Environment, The Hebrew University of Jerusalem,
Rehovot 7610001, Israel; yaa.amoah@mail.huji.ac.il
* Correspondence: moshesh@agri.gov.il

Table S1. Bacterial strains and their origins.

Bacteria	Strain	Genotype	Description	Reference
B. subtilis	NCIB 3610	wild type (WT)	Undomesticated WT strain	[1]
	YC 121	P*tapA*-LacZ	Transcriptional fusion of *tapA* promoter to generate β-galactosidase	[2]
	YC 161	P*spank-gfp* in 3610,	Consistently produces a green fluorescent protein (GFP)	[3]
	RL3852	Δ*epsH* in 3610	Matrix mutant lacking the ability to form biofilms	[4]
	SB505	Δ*tasA* in 3610	Matrix mutant lacking the ability to form biofilms	[5]

Table S2. Fibers used and their origin.

Fiber	Source	Country
Chickpea Fiber (CPF)	ChickP	Rehovot, Israel
Wheat Fiber (WF)	GSK	Warren, New Jersey, USA
Cellulose Fiber (CF)	Store-bought	Rehovot, Israel

Preparation of CPF

CPF, as used in this study, was prepared as follows.

Chickpea grains were processed for the extraction of proteins and oils. After the extraction, starch and fibers in the residues were physically separated based on densities. The resultant fibers from this process were the substrate labeled chickpea fiber, CPF.

References

1. Branda, S.S.; González-Pastor, J.E.; Ben-Yehuda, S.; Losick, R.; and Kolter, R. Fruiting Body Formation by *Bacillus subtilis*. *Proc. Natl. Acad. Sci. USA* **2001**, *98*, 11621–11626, doi:10.1073/PNAS.191384198.
2. Chai, Y.; Chu, F.; Kolter, R.; Losick, R. Bistability and Biofilm Formation in *Bacillus subtilis*. *Mol. Microbiol.* **2008**, *67*, 254–263, doi:10.1111/J.1365-2958.2007.06040.X.
3. Chai, Y.; Norman, T.; Kolter, R.; Losick, R. Evidence that metabolism and chromosome copy number control mutually exclusive cell fates in *Bacillus subtilis*. *EMBO Rep.* **2011**, *30*, 1402–1413, doi:10.1038/emboj.2011.36.
4. Branda, S.S.; Chu, F.; Kearns, D.B.; Losick, R.; Kolter, R.A. Major Protein Component of the *Bacillus subtilis* Biofilm Matrix. *Mol. Microbiol.* **2006**, *59*, 1229–1238, doi:10.1111/J.1365-2958.2005.05020.X.
5. Kearns, D.B.; Chu, F.; Rudner, R.; and Losick, R. Genes Governing Swarming in *Bacillus subtilis* and Evidence for a Phase Variation Mechanism Controlling Surface Motility. *Mol. Microbiol.* **2004**, *52*, 357–369, doi:10.1111/J.1365-2958.2004.03996.X
